# Genome-Wide Identification of Papain-Like Cysteine Proteases in *Gossypium hirsutum* and Functional Characterization in Response to *Verticillium dahliae*

**DOI:** 10.3389/fpls.2019.00134

**Published:** 2019-02-20

**Authors:** Shuqin Zhang, Zhongping Xu, Heng Sun, Longqing Sun, Muhammad Shaban, Xiyan Yang, Longfu Zhu

**Affiliations:** National Key Laboratory of Crop Genetic Improvement, Huazhong Agricultural University, Wuhan, China

**Keywords:** *Gossypium hirsutum*, papain-like cysteine proteases, phylogenetic analysis, gene expression, *Verticillium dahliae*

## Abstract

Cotton, a natural fiber producing crop of huge importance, is often prone to attack of *Verticillium dahliae*. Papain-like cysteine proteases (PLCPs) constitute a large family in plants and were proposed to involve in plant defense against pathogen attack in a number of studies. However, there is no detailed characterization of *PLCP* genes in cotton against infection of *V. dahliae*. In this study, we carried out a genome-wide analysis in cotton and identified seventy-eight PLCPs, which were divided into nine subfamilies based on their evolution phylogeny: RD21 (responsive to desiccation 21), CEP (cysteine endopeptidase), XCP (xylem cysteine peptidase), XBCP3 (xylem bark cysteine peptidase 3), THI, SAG12 (senescence-associated gene 12), RD19 (responsive to desiccation 19), ALP (aleurain-like protease) and CTB (cathepsin B-like). Genes in each subfamily exhibit a similar structure and motif composition. The expression patterns of these genes in different organs were examined, and subfamily RD21 was the most abundant in these families. Expression profiles under abiotic stress showed that thirty-five *PLCP* genes were induced by multiple stresses. Further transcriptome analysis showed that sixteen *PLCP* genes were up-regulated in response to *V. dahliae* in cotton. Among those, *GhRD21-7* showed a higher transcription level than most other *PLCP* genes. Additionally, over-expression of *GhRD21-7* led to enhanced resistance and RNAi lines were more susceptible to *V. dahliae* in cotton. Our results provide valuable information for future functional genomic studies of *PLCP* gene family in cotton.

## Introduction

Papain-like cysteine proteases (PLCPs), which belong to the family C1A of clan CA, are a class of proteolytic enzymes, with a catalytic cysteine as a nucleophile during proteolysis (Rawlings et al., 2010). PLCPs are widely found in virus, bacteria, yeast, protozoa, plants, and animals (Rawlings et al., 1992, 2010; Enenkel and Wolf, 1993; Kantyka et al., 2011; Novinec and Lenarcic, 2013). PLCPs are structurally characterized by a typical papain-like fold domain: an α-helix and β-sheet domain (Turk et al., 2001). Both domains are linked to each other, and the catalytic triad Cys-His-Asn forms at the two-domain interface (Turk et al., 2001). PLCPs are synthesized as preproproteases which contain a signal peptide, an auto-inhibitory pro-domain and a mature protease domain (Beers et al., 2004). After cleaving off an inhibitory pro-domain, PLCPs become active through self-processing or with the aid of processing enzymes. The first classification of 138 plant PLCPs are grouped into eight subfamilies primarily based on their structural characteristics (Beers et al., 2004). Later, 723 plant PLCPs were divided into nine classes according to their homology and domain architecture (Richau et al., 2012).

Recently, accumulating evidences have revealed that PLCPs play important roles in a variety of processes, including seed germination (Kiyosaki et al., 2010), anther development (Lee et al., 2004; Li et al., 2006; Zhang et al., 2009, 2014), defense against pathogens (Shindo and Van der Hoorn, 2008; van der Hoorn, 2008; Misas-Villamil et al., 2016), and response to insect attack (Pechan et al., 2002; Konno et al., 2004) or abiotic stresses (Khanna-Chopra et al., 1999). PLCPs are prominent enzymes in the plant apoplast and are key enzymes in the regulation of programmed cell death (PCD) (McLellan et al., 2009; Ge et al., 2016; Balakireva and Zamyatnin, 2018). PCD is a tightly regulated biological process which functions in many aspects of plant development and survival to multiple stresses. Depletion of PLCPs hampers plant immunity (Bernoux et al., 2008; Kaschani et al., 2010; Bozkurt et al., 2011; Shindo et al., 2012). Meanwhile, PLCPs are often targeted by a variety of unrelated pathogen-derived effectors (Kaschani et al., 2010; Bozkurt et al., 2011; Mueller et al., 2013; Clark et al., 2018). PLCPs also act as co-receptors and triggers immune response (Krüger et al., 2002).

Cotton (*Gossypium* spp.) is the most important cash crop that provides natural fiber for textile industry and oil for edible purposes. But during growth period it is greatly affected by various microbial pathogens and extreme environments. *Verticillium wilt*, a fungal disease causes a significant economic loss by greatly affecting cotton yield and fiber quality. This is a vascular disease and caused by soil-born pathogen *Verticillium dahlia.* The pathogen infects cotton roots, spread in vascular bundles, and caused wilting, necrosis, defoliation and even plant death under high severity. It has long been a challenge for cotton growth and demands enormous research to manage it efficiently. Previously various studies has shown that PLCPs are involved in plant defense against different pathogens, i.e., in tomato (*Lycopersicon esculentum* Mill.), the RCR3 (required for *Cladosporium* resistance 3) confers resistance to *Cladosporium fulvum*, and the activity is inhibited by *C. fulvum* effector Avr2 which activates Cf-2 (*C. fulvum* resistance gene-2) mediated immune responses (Rooney and Wit, 2005). However, the PLCP family in cotton is largely unknown, and the function of *PLCP* genes in response to *V. dahliae* infection has not been considered previously. Herein, we performed genome-wide screening and comprehensively analyzed the expression patterns of *PLCP* genes. We also determined that overexpression of one *PLCP* gene in cotton leads to enhanced resistance and RNAi lines were more susceptible to *V. dahliae.*

## Materials and Methods

### Identification of Papain-Like Cysteine Proteases in *G. hirsutum*

The available genome sequence of *G. hirsutum* was downloaded from cottongene^1^. Initial identification of PLCPs was carried out using HMMER^2^ against the Pfam^3^ Peptidase_C1 domain (PF00112) with default settings. The identified PLCPs were analyzed manually using the SMART^4^ and CDD^5^ databases for the presence of domains.

### Multiple Sequence Alignments and Phylogenetic Analysis

The ClustalX 1.83 was used for multiple sequence alignment of candidate PLCP sequences. We removed some sequences: (1) not containing the CWAF sequence; (2) Database entries with a N-terminal distance of less than 50 amino acids (aa); (3) Database entries with a C-terminal distance of less than 150 aa. Neighbor Joining phylogenetic tree was constructed using MEGA 6 (Molecular Evolutionary Genetics Analysis 6) software with 1000 bootstrap values. The predicted molecular weight (Mw) and isoelectric points (pI) of PLCPs were calculated using the online ExPASy program^6^.

### Gene Structure, Chromosome Distribution, and Gene Synteny Analysis of *PLCP* Genes

The organization of exon/intron was visualized using the online Gene Structure Display Server 2.0^7^ (GSDS, V.2) (Guo et al., 2007). MEME Suite^8^ (Bailey et al., 2006) was employed for identification of conserved motifs, and the optimized parameters were as follows: the number of motifs, 15; and the optimum width of each motif, between 6 and 50 residues.

Mapchart 2.2 software was used to map *PLCP* genes on the *G. hirsutum* chromosomes. Genome synteny was performed as described previously (Sun et al., 2017). The homologous gene pairs were visualized using circos package (version 0.4.4). The non-synonymous (dN) and synonymous (dS) substitution rates were calculated between the At (A subgenome, with lower-case “t” denoting tetraploid) and Dt (D subgenome, with lower-case “t” denoting tetraploid) to explore the evolutionary dynamics and selection pressure (Yang, 2007). The ratio dN/dS > 1 means positive selection, dN/dS = 1 means neutral selection, dN/dS < 1 means negative selection. The Maximum Likelihood (PAML) yn00 program with the GMYN method was used to calculate the ratio of non-synonymous to synonymous for the homologous gene pairs.

### Gene Expression Analysis of *PLCP* Genes

Expression data for *PLCP* genes was obtained from transcriptome data. These data sets corresponded to expression abundances of TM-1 (The allotetraploid cotton *G. hirsutum* L. acc. Texas Marker-1) in various tissues and stresses (Zhang et al., 2015) and in response to *V. dahliae* inoculation (Zhang et al., 2018). The expression values were normalized by Genesis software and showed by heatmap (Sturn et al., 2002).

### Plant Material and Fungal Pathogen Inoculation

*G. hirsutum* cv YZ1 cotton plants and transgenic lines of *Gh_RD21-7* derived from YZ1 were used in this study. For disease assays, plants were grown in a culture room with 16 h day/8 h night cycle at 25°C.

*V. dahliae* strain V991 was cultured on potato dextrose agar medium at 25°C for 5 days and highly activated hyphae were collected and cultivated in Czapek’s mediums at 25°C for 5 days. The final concentration of 10^6^ spore mL^-1^ was used for inoculation.

### Gene Cloning, Vector Construction, and Plant Transformation

The full-length of *Gh_RD21-7* sequence was obtained through RACE-PCR according to the SMART RACE cDNA amplification kit user manual (Clontech, United States) with YZ1 cDNA as the template. Full-length coding sequence was cloned into the Gateway vector pK2GW7.0 (Invitrogen, United States) to generate the overexpression vector. The conserved region of *Gh_RD21-7* was inserted into pHellsgate4 to generate the RNAi vector. The constructed vectors were transformed into *Agrobacterium tumefaciens* GV3101. The strain GV3101 containing different constructs were used to transform cotton (YZ1) plant via *A. tumefaciens-*mediated transformation (Jin et al., 2006).

### Southern Blotting and Expression Analysis

Genomic DNA was extracted from leaves using the plant genomic DNA kit DP305 (Tiangen Biotech, China). Approximately 20 μg of genomic DNA digested with HindIII (New England Biolabs, United States) was used for Southern blotting. Southern blotting was performed using the DIG-High Prime DNA Labeling and Detection Starter Kit II (Roche, Switzerland).

To confirm the expression level of transgenic lines of *Gh_RD21-7*, total RNA was extracted from cotton leaves following the protocol (Tu et al., 2007). Subsequently, the M-Mlv reverse transcript system was used for reverse transcription (Promega, United States). The quantitative real-time PCR (qRT-PCR) was performed using an ABI 7500 real time PCR system (Applied Biosystem, United States) as described previously (Hu et al., 2016). The fold changes were calculated using a comparative CT method (2^-ΔΔCt^) and the *GhUBQ7* gene was amplified as an internal control. Primers used in the study are listed in the Supplementary Table 1.

### Pathogen Infection and Disease Recovery Assay

To determine the resistance of different cotton lines in response to fungal pathogens, seedlings of wild-type and transgenic lines were supplied with Hoagland solution till to two-leaf-stage, and then inoculated with *V. dahliae* strain V991 with 10^6^ spores per liter for 1 h. After washing with water 3 times, inoculated seedlings were supplied with Hoagland solution again. At least 15 plants per treatment were used to score the disease leaves and at least three repetitions were performed. Disease index was calculated as described previously (Gao et al., 2013).

## Results

### Phylogenetic Analysis of Papain-Like Cysteine Proteases in *G. hirsutum*

We identified 78 PLCPs in *G. hirsutum* (Supplementary Table 2). These PLCPs varied in length from 278 to 606 aa, with Mw ranging from 31.2 to 67.5 kDa, and pI from 4.62 to 8.58 (Supplementary Table 3).

**FIGURE 1:**
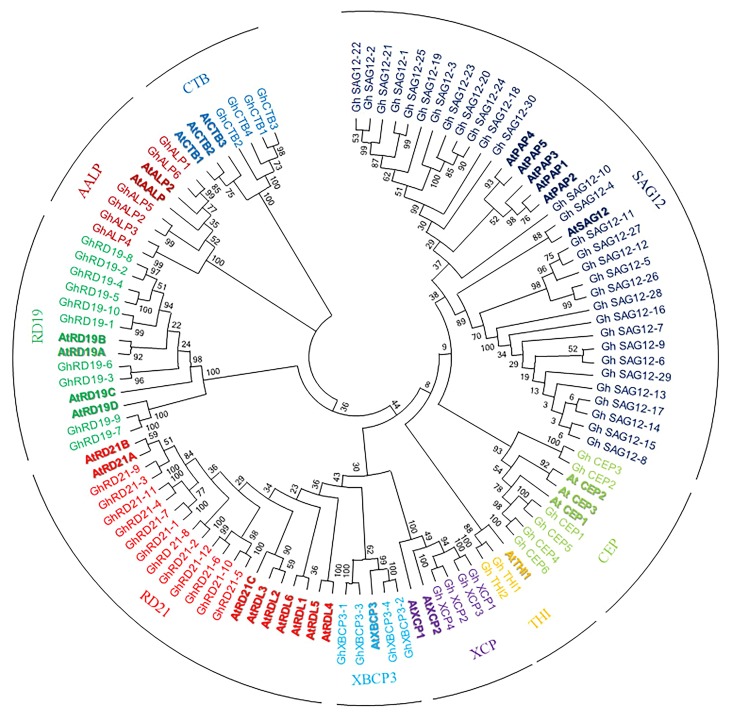
Phylogenetic analysis of upland cotton and *Arabidopsis* PLCPs. Phylogenetic tree was performed using MEGA6.

To investigate the evolutionary relationship among PLCPs, we used 78 PLCPs from *G. hirsutum* and 31 PLCPs from *Arabidopsis (Arabidopsis thaliana)* (Richau et al., 2012) for phylogenetic analysis (Supplementary Table 4). The phylogenetic tree separated PLCPs into nine different subfamilies (Figure 1), which was consistent with a previous study (Richau et al., 2012). We named nine subfamilies RD21 (responsive to desiccation 21), CEP (cysteine endopeptidase), XCP (xylem cysteine peptidase), XBCP3 (xylem bark cysteine peptidase 3), THI, SAG12 (senescence-associated gene 12), RD19 (responsive to desiccation 19), ALP (aleurain-like protease), and CTB (cathepsin B-like) according to keep the same naming system as previously reported by Richau et al. (2012).

PLCPs were not evenly distributed among the nine subfamilies. The subfamily SAG12 is the largest subfamily with 30 members, while the subfamily THI contains only 3 members. The subfamily RD21 and the subfamily RD19 contain 12 and 10 members, respectively. Both the subfamilies CEP and ALP contain six PLCPs. While, the subfamilies XCP, XBCP3, and CTB contain four PLCPs, respectively.

### Conserved Motif Distribution and Gene Structure Analysis

**FIGURE 2:**
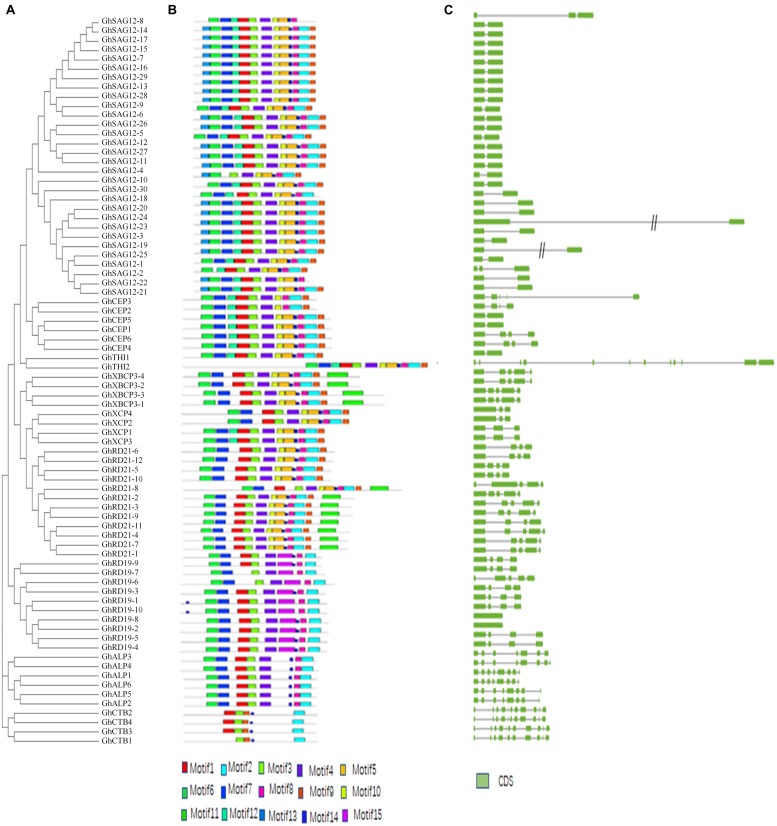
Structural and motif analysis of *PLCP* genes. **(A)** An unrooted phylogenetic tree constructed using MEGA6. **(B)** Distribution of 15 conserved motifs elucidated by MEME. **(C)** A graphic representation of exon-intron structures displayed using GSDS.

Multiple sequence alignment was employed to explore sequence features and MEME analysis was performed to predict distinct motifs. A total of 15 motifs named motifs 1–15, were finally identified (Figure 2A,B and Supplementary Figure 1). Expectedly, most of the closely related proteases share similar motif members in the same subfamily, suggesting that the protein architectures are remarkably conserved. Firstly, Motifs 13, 6, 7, and 12 are characterized as the auto-inhibitory pro-domain (PF08246). The sequence of motif 6 is ExxxRxxxFxxNxxxI/VxxxN with one mismatch in subfamilies RD21, CEP, XCP, XBCP3, THI, SAG12, and ALP, but the subfamily RD19 carries a conserved “EFRNAQ” motif instead of the “ERFNIN” motif. Secondly, motif 1, 3, 4, 10, 5, 14, 8, 2, and 9 are characterized as the Peptidase_C1 domain (PF00112). Motif 1, 8 and motif 2, which has a Cys, His and Asn catalytic sites, respectively. Catalytic triad (Cys-His-Asn) is conserved in upland cotton except GhSAG12-6 and GhSAG12-12 from subfamily SAG12, in which Gln and Arg are substituted with His in the active site. Motif 15 is specific to subfamily RD19. Thirdly, motif 11, which is included in subfamily XBCP3 and eight members in subfamily RD21, are characterized as the granulin-like domain (PF00396). On the other hand, the structures and motifs supported the results of phylogenetic analysis.

The gene structure of *PLCPs* was analyzed, and the results showed that these genes contain more than one intron, range from one to eleven (Figure 2C). Generally, a certain subfamily has a highly conserved exon-intron structure but different subfamilies have distinct structures. Subfamily RD21 features three or four introns. Subfamily CEP features one or four introns. Subfamily XCP features two introns. Subfamily XBCP3 features four introns. Subfamily SAG12 usually features one intron, except two *PLCP* genes. Subfamily RD19 most features three introns. Subfamily ALP most features seven introns. Subfamily CTB features nine introns.

### Chromosomal Location and Gene Synteny Analysis of *PLCP* Genes in *G. hirsutum*

**FIGURE 3:**
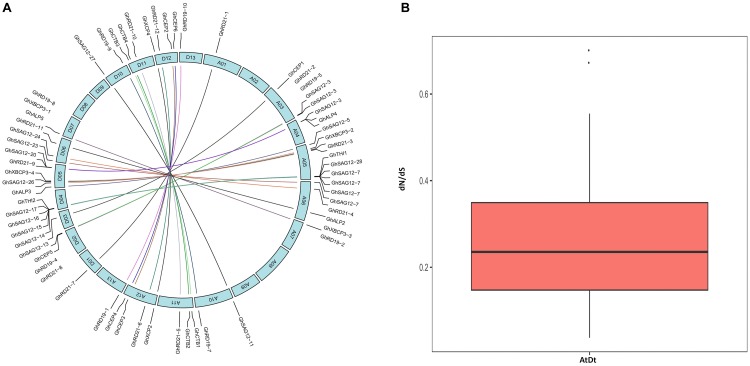
**(A)** The inter-genomic (At and Dt) synteny analysis of *PLCP* genes. Lines represent homologous genes that are distributed in syntenic blocks. **(B)** The ratio of nonsynonymous to synonymous substitutions (dN/dS) of *PLCP* genes in inter-genomic (At Dt).

Out of 78 *PLCP* genes, 76 genes were distributed on 21 chromosomes of *G. hirsutum*, while two genes (*GhSAG12-29* and *GhSAG12-30)* had no distinct chromosome location (Supplementary Table 5 and Supplementary Figure 2). These genes were unevenly distributed in the At and Dt. There were 36 genes in the At and 40 in the Dt. This suggests asymmetric evolution between the At and Dt. The distribution of 76 *PLCP* genes on each chromosome were not equal. Chromosomes A01, A03, A07, A10, A11, A13, D01, D02, D03, D06, D07, D10, D11, and D13 contained one to three *PLCP* genes, while the majority of *PLCP* genes (35 out of 76, 46%) were located on chromosomes A04, A05, D04, and D05. Furthermore, the *PLCP* genes in chromosomes A04, A05, D04, and D05 were primarily located on chromosome ends. In particular, closely related genes of the subfamily SAG12 were mainly located on chromosomes A04, A05, D04, and D05, suggesting that expansion of the *PLCP* gene family may have occurred via local or intra-chromosomal duplication. We further examined homologous gene pairs using a multiple homologous comparison analysis, which allowed us to identify 33 homologous gene pairs between the At and Dt (Figure 3A and Supplementary Table 6). The evolutionary dynamics and selection pressure were investigated by calculating the non-synonymous (dN) and synonymous (dS) substitution rates between the At and Dt. All of the dN is less than dS (dN/dS < 1), suggesting that purifying selection of *PLCP* genes has occurred in upland cotton (Figure 3B and Supplementary Table 6).

### Expression Profiles of *PLCPs* in Different Tissues

**FIGURE 4:**
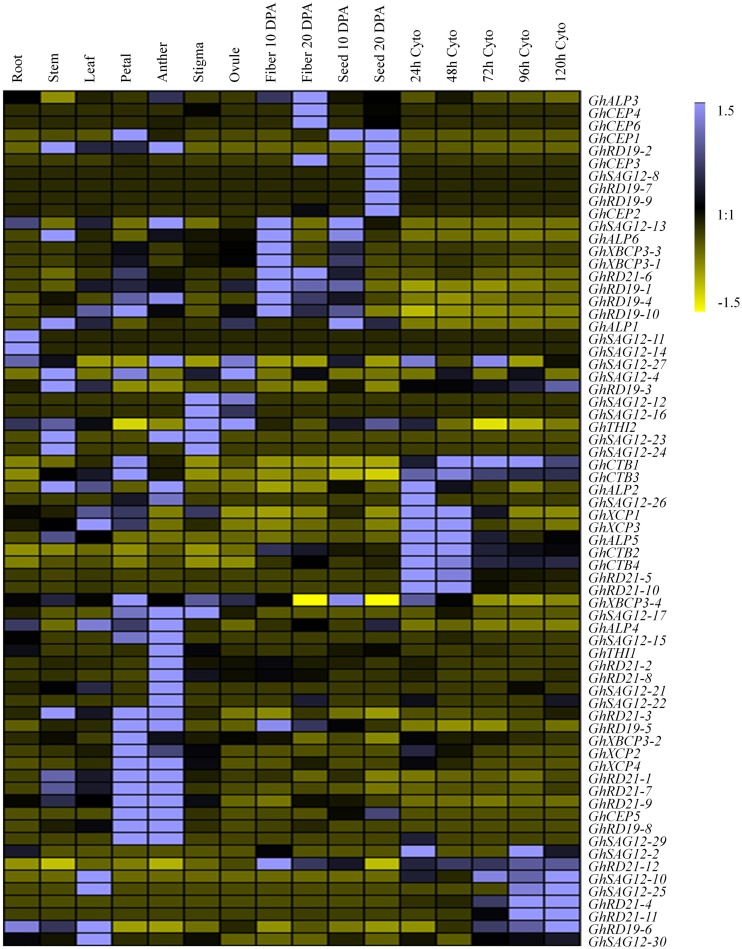
Expression pattern of *PLCP* genes in different tissues of *G*. *hirsutum*. DPA represent days post-anthesis. Cyto represent cotyledon. Color scale denotes FPKM normalized by Genesis software.

To explore the organ-specificity of *PLCP* genes, we examined the abundance of transcripts in different tissues of *G. hirsutum* TM-1, including root, stem, leaf, petal, anther, stigma, ovule, fiber, and seed at different development stages (10 days post-anthesis [dpa], and 20 dpa) and cotyledonary leaves at different stages (24, 48, 72, 96, and 120 h) (Zhang et al., 2015; Supplementary Table 7). According to the FPKM (Fragments Per Kilobase of transcript per Million base pairs sequenced) values, these *PLCP* genes were most abundant in petal and anther, followed by fiber (10 dpa), cotyledon (24 h), stem and seed (10 dpa), with relatively low expression in stigma, ovule and root. Among all the examined tissues, the subfamily RD21 composed the major *PLCP* expression level. In root, subfamily RD21 occupied about 30% of total *PLCP* expression level. In stigma, fiber (20 dpa) and cotyledon at different stages (24, 48 h), subfamily RD21 was also the most abundant (Figure 4).

Based on the expression pattern across nine subfamilies, the expression level was diverse. Subfamily RD21 contributed the most expression level, followed by subfamilies ALP, CEP, RD19, CTB, XBCP3. Subfamilies XCP, SAG12, and THI had low expression levels. Subfamily RD21 occupied about 25.4% of total *PLCP* expression level. In subfamily RD21, *GhRD21-7* expressed the most, and occupied about 17.4%. Particularly, subfamily SAG12 had the most members, but few of these was expressed.

### Expression Patterns of the *PLCP* Genes Under Abiotic Stresses

Given the possibility that cotton *PLCP* genes might play an important role in response to various environmental stresses, the expression patterns were investigated in response to heat, salt, cold, and polyethylene glycol (PEG), using public transcriptome datasets (Zhang et al., 2015; Supplementary Table 8). Thirty-five *PLCP* genes exhibited twofold expression changes compared with controls in at least one treatment. Moreover, all of these 35 *PLCP* genes were differentially expressed under heat or cold stresses (Supplementary Figure 3). There were 21, 10, 24, and 8 differentially expressed *PLCP* genes which were identified in heat, salt, cold, and PEG stress treatments, respectively. Further analysis showed that 19 *PLCP* genes were differentially expressed under at least two stress conditions. Interestingly, six genes (*GhRD21-5*, *GhXBCP3-4*, *GhRD21-10*, *GhALP2*, *GhRD21-7* and *GhRD19-6*) were identified only under heat and cold stress conditions.

### Expression Patterns of the *PLCP* Genes Under *V. dahliae* Infection

**FIGURE 5:**
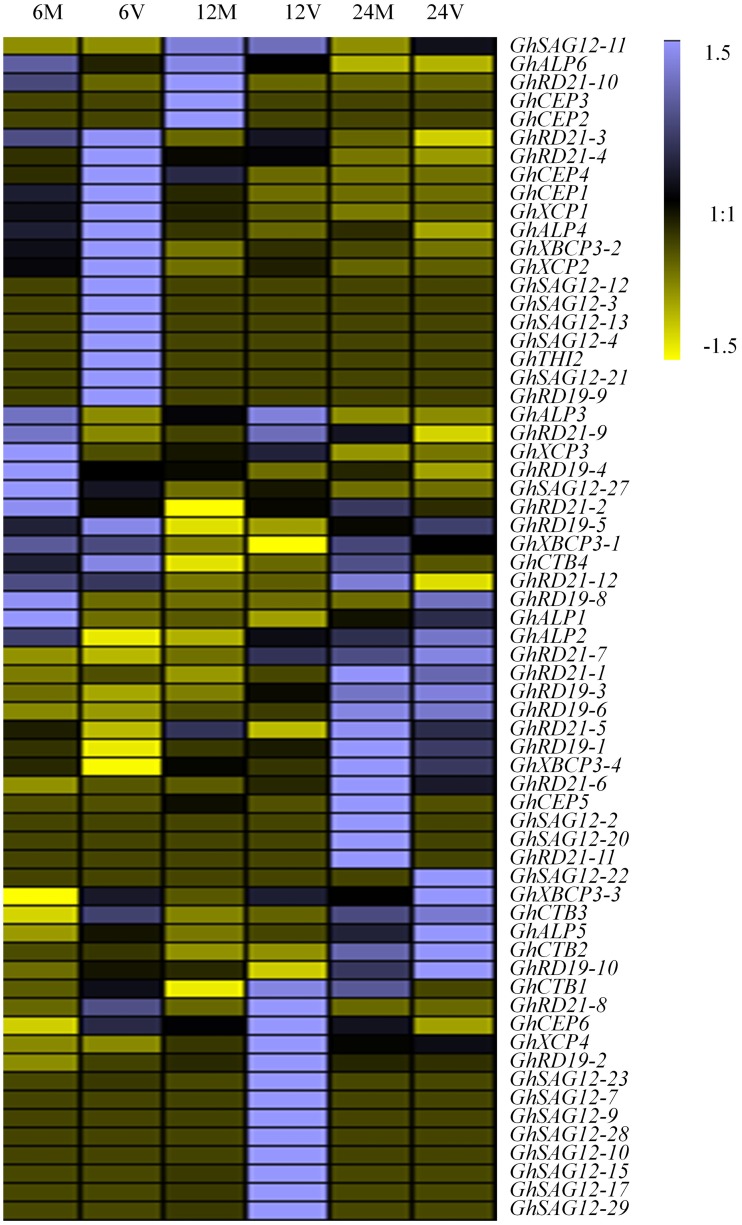
Expression analysis of *PLCP* genes under *V. dahliae* inoculation in *G*. *hirsutum* cv YZ1. 6M, 12M, 24M represent 6, 12, 24 h Mock 6V, 12V, 24V represent 6, 12, 24 h post V991 infection.

Considering potential roles of *PLCP* genes in pathogen defense, we investigated the transcript abundance of *PLCP* genes in response to *V. dahliae*, using transcriptomic data provided by Zhang et al. (2018) (Supplementary Table 9). Some *PLCP* genes expressed faintly or not expressed at the special moment. Twenty-nine *PLCP* genes with twofold expression changes were identified after *V. dahliae* inoculation (Figure 5). There were nine genes which were up-regulated at 6 h post infection [hpi], and six genes were up-regulated at 12 hpi, meanwhile one gene (*GhXCP2*) was up-regulated from 6 to 12 hpi. On the other hand, four genes were down-regulated at 6 and 24 hpi, respectively, and one gene (*GhALP6*) was down-regulated from 6 to 12 hpi. Interestingly, *GhRD21-4* and *GhCEP6* were up-regulated at 6 hpi but down-regulated at 24 hpi. *GhCTB4* was up-regulated at 12 hpi but down-regulated at 24 hpi. *GhALP3* was down-regulated at 6 hpi but up-regulated at 12 hpi. *GhRD21-7* exhibited a higher expression level than most other PLCP genes, it down-regulated at 6 hpi, but up-regulated at 12 hpi, and sustained at 24 hpi.

### Overexpressing *GhRD21-7* Enhances Plant Tolerance to *V. dahliae*

**FIGURE 6:**
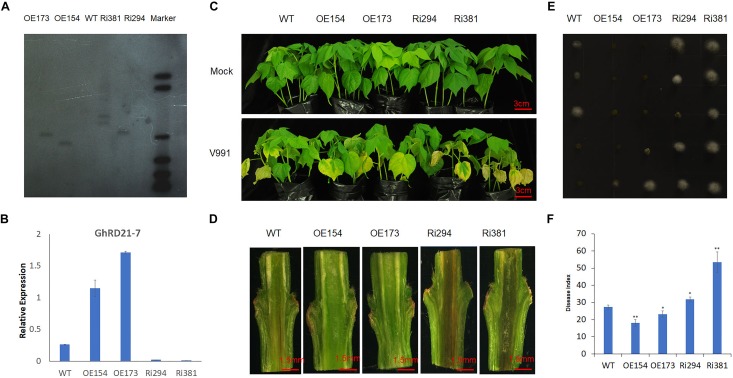
*GhRD21-7* is a positive regulator of cotton resistance to *V. dahliae.* **(A)** Southern blotting analysis in transgenic cotton lines. **(B)** Quantitative RT-PCR analysis of *GhRD21-7* expression level. **(C)** Disease symptoms in wild type, overexpression lines, and RNAi plants after inoculation with *V. dahliae* strain V991, and photographed 18 days after inoculation. **(D)** V991 hyphal growth in wild-type and transgenic plants after inoculation 19 days. **(E)** Fungal recovery assay, and photographed 3 days after inoculation. **(F)** Disease indices of wild-type and transgenic plants were determined after inoculation with V991 for 17 days. The values are the means ± standard error, *n* = 3. Statistical analyses were performed using Student’s *t*-test (^∗^*P* < 0.05, ^∗∗^*P* < 0.01). All the experiments were repeated at least three times with similar results.

It was found previously that *GhRD21-7* (*CysP*) was involved in cotton immune response to *V. dahliae* and induced by *V. dahliae* strain V991 at the early stage of infection (Xu et al., 2011). To elucidate the putative role of *PLCP* genes during cotton defense to *V. dahliae*, the overexpression and RNA interference (RNAi) were used to generate stable transgenic cotton lines by up-regulating or down-regulating the expression of *GhRD21-7*, respectively. We selected two independent overexpression lines of *GhRD21-7* (OE154 and OE173) and two independent RNAi lines (Ri294 and Ri381) based on Southern blotting and expression levels (Figure 6A,B). The different transgenic and wild-type cotton seedlings were inoculated with *V. dahliae* strain V991 at the three leaf-stage. Typical disease symptoms, including extensive chlorosis, wilting, and necrosis (Li et al., 2014; Hu et al., 2018), were much more severe in the RNAi lines, which indicates that knock-down of *GhRD21-7* compromise the resistance (Figure 6C). The corresponding vascular bundles, fungal recovery assay and disease index analysis also supported these results (Figure 6D–F). Therefore, over-expression of *GhRD21-7* improves tolerance to *V. dahliae* and down-expression of *GhRD21-7* enhances susceptibility to the fungus.

## Discussion

Genome-wide scan of PLCPs has been systematically carried out in *Arabidopsis* (Richau et al., 2012), rice (*Oryza sativa* L.) (Wang et al., 2018), citrus (*Citrus sinensis)* (Clark et al., 2018), castor bean (*Ricinus communis*) (Zou et al., 2018), physic nut (*Jatropha curcas*) (Zou et al., 2018), Carica papaya (Liu et al., 2018), and rubber (*Hevea brasiliensis*) (Zou et al., 2017). In the current study, a total of 78 PLCPs were identified in *G. hirsutum*. These PLCPs were grouped into nine subfamilies according to their phylogenetic clade and structure features, which were similar to previous studies (Richau et al., 2012). However, in different species, the numbers of PLCPs varied largely. *G. hirsutum* contains 78 members; *Arabidopsis* contains 31 members; rice contains 33 members; citrus contains 21 members; castor bean contains 26 members; physic nut contains 23 members; Carica papaya contains 33 members; rubber contains 43 members. Meanwhile, genes number in each subfamily is out of proportion among some plant species. Significant variations of gene number in each subfamily may result from whole genome duplication, tandem duplication and large-scale segmental duplication. Liu et al. (2018) revealed that tandem duplication played a significant role in affecting lineage-specific expansion of PLCPs in Carica papaya. The number of PLCPs in *G. hirsutum* is larger than in other species, and the reason may be that *G. hirsutum* is an allotetraploid species coming from two divergent A and D genomes (Wendel and Cronn, 2003; Wendel et al., 2012). Our synteny analysis led to identify 33 homologous gene pairs between At and Dt. Further through multiple homologous comparison analysis, we found 24 homologous gene pairs which show close evolution, such as exon-intron structures and conserved motifs. For selective pressure analysis of cotton *PLCP* genes, the purifying selecting acted as a primary force, this indicated that the ancestral functions might sustain in the evolutionary process of cotton. A similar classification of PLCPs was divided into four major groups: cathepsin L-, F-, H-, and B- like proteases according to their closest animal counterparts and the motif in N-terminal pre-domain. “ERFNIN” motif is a marker motif for cathepsin L- and H-like proteases, but does not exist in cathepsin B-like proteases (Coulombe et al., 1996; Kramer et al., 2017). “EFRNAQ” motif is typical for cathepsin F-like proteases (Kramer et al., 2017). According to this principle, subfamilies RD21, CEP, XCP, XBCP3, THI, and SAG12 which were associated with each other, were also called cathepsin L-like PLCPs. Subfamilies RD19, ALP, and CTB, distinct form subfamilies RD21, CEP, XCP, XBCP3, THI, and SAG12, called cathepsin F-like, cathepsin H-like and cathepsin B-like PLCPs, respectively. Granulins were originally described in the animal kingdom and had multiple biological roles (Bateman and Bennett, 2009). Some C-terminal extension (Cx5Cx5CCCx7Cx4CCx6CCx5CCx6Cx6C), consisting of a granulin-like domain was also detected in subfamilies RD21 and XBCP3. However, not all PLCPs of the subfamily RD21 contain a granulin domain. This implies that the granulin polymorphism evolved by loss-of-domain and the roles need to be further studied in the future (Richau et al., 2012).

Abiotic and biotic stresses seriously impact cotton growth and yield, more and more molecular mechanisms about cotton response to different stresses were discovered in recent years, but no PLCPs in cotton have been reported. In this study, a comparison of expression patterns of homologous genes verified that some of these genes exhibited similar responses toward abiotic treatment. For instance, *GhRD21-1/GhRD21-7*, *GhRD21-3/GhRD21-9*, *GhRD21-5/GhRD21-10*, and *GhRD19-5/GhRD19-4* showed similar responses to stress treatments. We identified 29 *PLCP* genes with twofold expression changes after *V. dahliae* invasion. There were six homologous pairs (*GhCEP1/GhCEP5*, *GhCEP4/GhCEP6*, *GhXCP2/GhXCP4*, *GhALP3/GhALP4*, *GhALP5/GhALP2*, *GhALP6/GhALP1*) within these genes, and both homologous genes showed different expression patterns. Gene expression patterns suggest that homologous genes had partially overlapping function, and the redundant functions of *PLCP* genes may contribute to protect the plant from various stress conditions. On the other hand, homologous *PLCP* genes also showed functional divergence, implying that *PLCP* genes may play an important role in driving evolutionary novelty and adaptation to new environments.

PLCPs have been reported to regulate plant immunity and responsible for defense against a board range of pathogens including fungi, bacteria, and oomycetes (Rooney and Wit, 2005; Lozano-Torres et al., 2012; Ilyas et al., 2015; Shindo et al., 2016). For example, in tomato, the SAG12 subfamily members, RCR3 and PIP1 (phytophthora inhibited protease 1), are targeted and contribute to the defense against oomycete pathogen (Rooney and Wit, 2005; Kaschani et al., 2010). In citrus, SAG12 subfamily exhibits an increased protein accumulation in *Candidatus Liberibacter* asiaticus infected trees, which suggests that PLCPs play an important role in defense response (Clark et al., 2018). After knocking out or silencing RD21 subfamily members, plants were more susceptible to necrotrophic fungal and oomycete pathogens (Kaschani et al., 2010; Bozkurt et al., 2011; Shindo et al., 2012). In maize (*Zea mays* L.), lacking of CP1A (cysteine protease1-likeA), CP1B (cysteine protease1-likeB), CP2 (core cysteine protease 2) and XCP2 result in increased susceptibility to *Ustilago maydis* (Mueller et al., 2013). Similarly, RD19 subfamily is required for RRS1-R (resistant to *Ralstonia solanacearum* 1-R) mediated resistance against the bacterial type III effector PopP2 (Pseudomonas outer protein P2) in *Arabidopsis* (Bernoux et al., 2008). Silencing of subfamily cathepsin B attenuates the hypersensitive response (HR) and increases susceptibility to the non-host bacterial pathogens (McLellan et al., 2009). The mechanisms underlying PLCP-guided defense can work on multiple levels. PLCPs may straightly catalyze pathogen components to prevent pathogen growth or cleave host peptide to elicit defense responses (Misas-Villamil et al., 2016; Gumtow et al., 2017; Balakireva and Zamyatnin, 2018). Although PLCPs are often targeted by fungi, bacteria and oomycetes effectors, none of these effectors shares sequence similarities, indicating that PLCPs subfamily are an important kind of regulators for plant defense.

*V. dahliae* is currently considered to be the most destructive disease of cotton worldwide (Xia et al., 1998; Bowman, 1999). Only one resistance (R) gene (*Ve1*) which contributes efficient resistance against *V. dahliae* race1, was isolated from tomato (Kawchuk et al., 2001). However, over-expression of *Ve1* in cotton did not confer tolerance to *V. dahliae* strain V991 (Liu et al., 2014). Therefore, some genes with broad-spectrum resistance should be identified. In this study, we found *GhRD21-7 was induced by V. dahliae* from 12 to 24 hpi. Previous study also found *GhRD21-7* (*CysP*) was induced by *V. dahliae* (Xu et al., 2011). Therefore, we generated stable transgenic cotton lines. Overexpression of *GhRD21-7* led to enhanced tolerance of *V. dahliae* and suppression of *GhRD21-7* increased susceptibility to *V. dahliae*. C14, the subfamily RD21 member in tomato, was targeted by effector Avrblb2 of *Phytophthora infestans*, which prevented C14 secretion into the apoplast and blocked the plant immunity (Bozkurt et al., 2011). CP1A and CP1B were subfamily RD21 members in maize, whose activity were inhibited by *Ustilago maydis* effector Pit2 (protein involved in tumors 2). The activity of CP1A and CP1B was related to salicylic-acid-associated plant defense (Mueller et al., 2013). Here, we carried out a genome-wide scan of PLCPs in *G. hirsutum* and found that *GhRD21-7* should act as a regulator to improve tolerance to *V. dahliae*. Further experiments are required to reveal the mechanisms by which *GhRD21-7* contribute to cotton defense signaling and enhance immune response to *V. dahliae*.

## Author Contributions

SZ designed and carried out the experiments, and also wrote the manuscript. ZX and HS contributed to bioinformatics analysis. LS generated the transgenic plants. XY, MS, and LZ directed and revised the manuscript. All the authors read and approved the final manuscript.

## Conflict of Interest Statement

The authors declare that the research was conducted in the absence of any commercial or financial relationships that could be construed as a potential conflict of interest.

## Footnotes

1 http://www.cottongen.org/

2 http://www.hmmer.org/

3 http://pfam.xfam.org/

4 http://smart.embl-heidelberg.de/

5 https://www.ncbi.nlm.nih.gov/Structure/cdd/wrpsb.cgi

6 http://web.expasy.org/protparam/

7 http://gsds.cbi.pku.edu.cn/

8 http://meme-suite.org/index.html

## References

[B1] Bailey T. L. Williams N. Misleh C. Li W. W. (2006). MEME: discovering and analyzing DNA and protein sequence motifs. *Nucleic Acids Res.* 34 W369–W373. 10.1093/nar/gkl198 16845028 PMC1538909

[B2] Balakireva A. V. Zamyatnin A. A. (2018). Indispensable role of proteases in plant innate immunity. *Int. J. Mol. Sci.* 19:E629. 10.3390/ijms19020629 29473858 PMC5855851

[B3] Bateman A. Bennett H. P. (2009). The granulin gene family: from cancer to dementia. *Bioessays* 31 1245–1254. 10.1002/bies.200900086 19795409

[B4] Beers E. P. Jones A. M. Dickerman A. W. (2004). The S8 serine, C1A cysteine and A1 aspartic protease families in Arabidopsis. *Phytochemistry* 65 43–58. 10.1016/j.phytochem.2003.09.005 14697270

[B5] Bernoux M. Timmers T. Jauneau A. Briere C. de Wit P. J. Marco Y. (2008). RD19, an Arabidopsis cysteine protease required for RRS1-R-mediated resistance, is relocalized to the nucleus by the *Ralstonia solanacearum* PopP2 effector. *Plant Cell* 20 2252–2264. 10.1105/tpc.108.058685 18708476 PMC2553607

[B6] Bowman D. T. (1999). Public cotton breeders–do we need them? *J. Cotton Sci.* 3 139–152. 16263937

[B7] Bozkurt T. O. Schornack S. Win J. Shindo T. Ilyas M. Oliva R. (2011). *Phytophthora infestans* effector AVRblb2 prevents secretion of a plant immune protease at the haustorial interface. *Proc. Natl. Acad. Sci. U.S.A.* 108 20832–20837. 10.1073/pnas.1112708109 22143776 PMC3251060

[B8] Clark K. Franco J. Y. Schwizer S. Pang Z. Hawara E. Liebrand T. W. H. (2018). An effector from the Huanglongbing-associated pathogen targets citrus proteases. *Nat. Commun.* 9:1718. 10.1038/s41467-018-04140-9 29712915 PMC5928222

[B9] Coulombe R. Grochulski P. Sivaraman J. Ménard R. Mort J. S. Cygler M. (1996). Structure of human procathepsin L reveals the molecular basis of inhibition by the prosegment. *EMBO J.* 15 5492–5503. 10.1002/j.1460-2075.1996.tb00934.x 8896443 PMC452294

[B10] Enenkel C. Wolf D. H. (1993). BLH1 codes for a yeast thiol aminopeptidase, the equivalent of mammalian bleomycin hydrolase. *J. Biol. Chem.* 268 7036–7043. 8463237

[B11] Gao W. Long L. Zhu L. F. Xu L. Gao W. H. Sun L. Q. (2013). Proteomic and virus-induced gene silencing (VIGS) analyses reveal that gossypol, brassinosteroids, and jasmonic acid contribute to the resistance of cotton to *Verticillium dahliae*. *Mol. Cell. Proteomics* 12 3690–3703. 10.1074/mcp.M113.031013 24019146 PMC3861717

[B12] Ge Y. Cai Y. M. Bonneau L. Rotari V. Danon A. McKenzie E. A. (2016). Inhibition of cathepsin B by caspase-3 inhibitors blocks programmed cell death in Arabidopsis. *Cell Death Differ.* 23 1493–1501. 10.1038/cdd.2016.34 27058316 PMC5072426

[B13] Gumtow R. Wu D. Uchida J. Tian M. (2017). A *Phytophthora palmivora* extracellular cystatin-like protease inhibitor targets papain to contribute to virulence on papaya. *Mol. Plant Microbe Interact.* 31 363–373. 10.1094/MPMI-06-17-0131-FI 29068239

[B14] Guo A. Y. Zhu Q. H. Chen X. Luo J. C. (2007). GSDS: a gene structure display server. *Hereditas* 29 1023–1026. 10.1360/yc-007-1023 17681935

[B15] Hu H. He X. Tu L. Zhu L. Zhu S. Ge Z. (2016). GhJAZ2 negatively regulates cotton fiber initiation by interacting with the R2R3-MYB transcription factor GhMYB25-like. *Plant J.* 88 921–935. 10.1111/tpj.13273 27419658

[B16] Hu Q. Min L. Yang X. Jin S. Zhang L. Li Y. (2018). Laccase GhLac1 modulates broad-spectrum biotic stress tolerance via manipulating phenylpropanoid pathway and jasmonic acid synthesis. *Plant Physiol.* 176 1808–1823. 10.1104/pp.17.01628 29229698 PMC5813555

[B17] Ilyas M. Horger A. C. Bozkurt T. O. van den Burg H. A. Kaschani F. Kaiser M. (2015). Functional divergence of two secreted immune proteases of tomato. *Curr. Biol.* 25 2300–2306. 10.1016/j.cub.2015.07.030 26299516

[B18] Jin S. Zhang X. Nie Y. Guo X. Liang S. Zhu H. (2006). Identification of a novel elite genotype for in vitro culture and genetic transformation of cotton. *Biol. Plant.* 50 519–524. 10.1007/s10535-006-0082-5

[B19] Kantyka T. Shaw L. N. Potempa J. (2011). Papain-like proteases of *Staphylococcus aureus*. *Adv. Exp. Med. Biol.* 712 1–14. 10.1007/978-1-4419-8414-2_1 21660655

[B20] Kaschani F. Shabab M. Bozkurt T. Shindo T. Schornack S. Gu C. (2010). An effector-targeted protease contributes to defense against *Phytophthora infestans* and is under diversifying selection in natural hosts. *Plant Physiol.* 154 1794–1804. 10.1104/pp.110.158030 20940351 PMC2996022

[B21] Kawchuk L. M. Hachey J. Lynch D. R. Kulcsar F. Van R. G. Waterer D. R. (2001). Tomato Ve disease resistance genes encode cell surface-like receptors. *Proc. Natl. Acad. Sci. U.S.A.* 98 6511–6515. 10.1073/pnas.091114198 11331751 PMC33499

[B22] Khanna-Chopra R. Srivalli B. Ahlawat Y. S. (1999). Drought induces many forms of cysteine proteases not observed during natural senescence. *Biochem. Biophys. Res. Commun.* 255:324. 10.1006/bbrc.1999.0195 10049707

[B23] Kiyosaki T. Matsumoto I. Asakura T. Funaki J. Kuroda M. Misaka T. (2010). Gliadain, a gibberellin-inducible cysteine proteinase occurring in germinating seeds of wheat, *Triticum aestivum* L., specifically digests gliadin and is regulated by intrinsic cystatins. *FEBS J.* 274 1908–1917. 10.1111/j.1742-4658.2007.05749.x 17371549

[B24] Konno K. Hirayama C. Nakamura M. Tateishi K. Tamura Y. Hattori M. (2004). Papain protects papaya trees from herbivorous insects: role of cysteine proteases in latex. *Plant J.* 37 370–378. 10.1046/j.1365-313X.2003.01968.x 14731257

[B25] Kramer L. Turk D. Turk B. (2017). The future of cysteine cathepsins in disease management. *Trends Pharmacol. Sci.* 38 873–898. 10.1016/j.tips.2017.06.003 28668224

[B26] Krüger J. Thomas C. M. Golstein C. Dixon M. S. Smoker M. Tang S. (2002). A tomato cysteine protease required for Cf-2-dependent disease resistance and suppression of autonecrosis. *Science* 296 744–747. 10.1126/science.1069288 11976458

[B27] Lee S. Jung K. H. An G. Chung Y. Y. (2004). Isolation and characterization of a rice cysteine protease gene, OsCP1, using T-DNA gene-trap system. *Plant Mol. Biol.* 54 755–765. 10.1023/B:PLAN.0000040904.15329.29 15356393

[B28] Li C. He X. Luo X. Xu L. Liu L. Min L. (2014). Cotton WRKY1 mediates the plant defense-to-development transition during infection of cotton by *Verticillium dahliae* by activating JASMONATE ZIM-DOMAIN1 expression. *Plant Physiol.* 166 2179–2194. 10.1104/pp.114.246694 25301887 PMC4256851

[B29] Li N. Zhang D. S. Liu H. S. Yin C. S. Li X. X. Liang W. Q. (2006). The rice tapetum degeneration retardation gene is required for tapetum degradation and anther development. *Plant Cell* 18 2999–3014. 10.1105/tpc.106.044107 17138695 PMC1693939

[B30] Liu J. Sharma A. Niewiara M. J. Singh R. Ming R. Yu Q. (2018). Papain- like cysteine proteases in *Carica papaya*: lineage-specific gene duplication and expansion. *BMC Genomics* 19:26. 10.1186/s12864-017-4394-y 29306330 PMC5756445

[B31] Liu L. Zhang W. Zhou Y. Miao Y. Xu L. Liu M. (2014). Resistance of cotton and tomato to *Verticillium dahliae* from cotton is independent on Ve1. *Sci. Sin.* 44 803–814. 10.1360/052014-90

[B32] Lozano-Torres J. L. Wilbers R. H. P. Gawronski P. Boshoven J. C. Finkers-Tomczak A. Cordewener J. H. G. (2012). Dual disease resistance mediated by the immune receptor Cf-2 in tomato requires a common virulence target of a fungus and a nematode. *Proc. Natl. Acad. Sci. U.S.A.* 109 10119–10124. 10.1073/pnas.1202867109 22675118 PMC3382537

[B33] McLellan H. Gilroy E. M. Yun B. W. Birch P. R. Loake G. J. (2009). Functional redundancy in the Arabidopsis Cathepsin B gene family contributes to basal defence, the hypersensitive response and senescence. *New Phytol.* 183 408–418. 10.1111/j.1469-8137.2009.02865.x 19453434

[B34] Misas-Villamil J. C. van der Hoorn R. A. Doehlemann G. (2016). Papain-like cysteine proteases as hubs in plant immunity. *New Phytol.* 212 902–907. 10.1111/nph.14117 27488095

[B35] Mueller A. N. Ziemann S. Treitschke S. Assmann D. Doehlemann G. (2013). Compatibility in the *Ustilago maydis*-maize interaction requires inhibition of host cysteine proteases by the fungal effector Pit2. *PLoS Pathog.* 9:e1003177. 10.1371/journal.ppat.1003177 23459172 PMC3573112

[B36] Novinec M. Lenarcic B. (2013). Papain-like peptidases: structure, function, and evolution. *Biomol. Concepts* 4 287–308. 10.1515/bmc-2012-0054 25436581

[B37] Pechan T. Cohen A. Williams W. P. Luthe D. S. (2002). Insect feeding mobilizes a unique plant defense protease that disrupts the peritrophic matrix of caterpillars. *Proc. Natl. Acad. Sci. U.S.A.* 99 13319–13323. 10.1073/pnas.202224899 12235370 PMC130631

[B38] Rawlings N. D. Barrett A. J. Bateman A. (2010). MEROPS: the peptidase database. *Nucleic Acids Res.* 38 D227–D233. 10.1093/nar/gkp971 19892822 PMC2808883

[B39] Rawlings N. D. Pearl L. H. Buttle D. J. (1992). The baculovirus *Autographa californica* nuclear polyhedrosis virus genome includes a papain-like sequence. *Biol. Chem. Hoppe Seyler* 373 1211–1216. 10.1515/bchm3.1992.373.2.1211 1363350

[B40] Richau K. H. Kaschani F. Verdoes M. Pansuriya T. C. Niessen S. Stuber K. (2012). Subclassification and biochemical analysis of plant papain-like cysteine proteases displays subfamily-specific characteristics. *Plant Physiol.* 158 1583–1599. 10.1104/pp.112.194001 22371507 PMC3320171

[B41] Rooney H. C. E. Wit P. J. G. M. D. (2005). Cladosporium Avr2 inhibits tomato Rcr3 protease required for Cf-2-dependent disease resistance. *Science* 308 1783–1786. 10.1126/science.1111404 15845874

[B42] Shindo T. Kaschani F. Fan Y. Kovács J. Fang T. Kourelis J. (2016). Screen of non-annotated small secreted proteins of *Pseudomonas* syringae reveals a virulence factor that inhibits tomato immune proteases. *PLoS Pathog.* 12:e1005874. 10.1371/journal.ppat.1005874 27603016 PMC5014320

[B43] Shindo T. Misas-Villamil J. C. Horger A. C. Song J. van der Hoorn R. A. (2012). A role in immunity for Arabidopsis cysteine protease RD21, the ortholog of the tomato immune protease C14. *PLoS One* 7:e29317. 10.1371/journal.pone.0029317 22238602 PMC3253073

[B44] Shindo T. Van der Hoorn R. A. (2008). Papain-like cysteine proteases: key players at molecular battlefields employed by both plants and their invaders. *Mol. Plant Pathol.* 9 119–125. 10.1111/j.1364-3703.2007.00439.x 18705889 PMC6640327

[B45] Sturn A. Quackenbush J. Trajanoski Z. (2002). Genesis: cluster analysis of microarray data. *Bioinformatics* 18 207–208. 10.1093/bioinformatics/18.1.207 11836235

[B46] Sun H. Chen L. Li J. Hu M. Ullah A. He X. (2017). The JASMONATE ZIM-domain gene family mediates JA signaling and stress response in cotton. *Plant Cell Physiol.* 58 2139–2154. 10.1093/pcp/pcx148 29036515

[B47] Tu L. L. Zhang X. L. Liang S. G. Liu D. Q. Zhu L. F. Zeng F. C. (2007). Genes expression analyses of sea-island cotton (*Gossypium barbadense* L.) during fiber development. *Plant Cell Rep.* 26 1309–1320. 10.1007/s00299-007-0337-4 17377794

[B48] Turk V. Turk B. Turk D. (2001). Lysosomal cysteine proteases: facts and opportunities. *EMBO J.* 20 4629–4633. 10.1093/emboj/20.17.4629 11532926 PMC125585

[B49] van der Hoorn R. A. (2008). Plant proteases: from phenotypes to molecular mechanisms. *Annu. Rev. Plant Biol.* 59 191–223. 10.1146/annurev.arplant.59.032607.092835 18257708

[B50] Wang W. Zhou X. M. Xiong H. X. Mao W. Y. Zhao P. Sun M. X. (2018). Papain-like and legumain-like proteases in rice: genome-wide identification, comprehensive gene feature characterization and expression analysis. *BMC Plant Biol.* 18:87. 10.1186/s12870-018-1298-1 29764367 PMC5952849

[B51] Wendel J. F. Cronn R. C. (2003). Polyploidy and the evolutionary history of cotton. *Adv. Agron.* 78 139–186. 10.1016/S0065-2113(02)78004-8

[B52] Wendel J. F. Flagel L. E. Adams K. L. (2012). “Jeans, genes, and genomes: cotton as a model for studying polyploidy,” in *Polyploidy and Genome Evolution*, eds Pamela S. S. Douglas E. S. (Berlin: Springer-Verlag), 181–207.

[B53] Xia Z. Achar P. N. Gu B. (1998). Vegetative compatibility groupings of *Verticillium dahliae* from cotton in mainland China. *Eur. J. Plant Pathol.* 104 871–876. 10.1023/A:1008628209867

[B54] Xu L. Zhu L. Tu L. Guo X. Long L. Sun L. (2011). Differential gene expression in cotton defence response to *Verticillium dahliae* by SSH. *J. Phytopathol.* 159 606–615. 10.1111/j.1439-0434.2011.01813.x

[B55] Yang Z. (2007). PAML 4: phylogenetic analysis by maximum likelihood. *Mol. Biol. Evol.* 24 1586–1591. 10.1093/molbev/msm088 17483113

[B56] Zhang D. Liu D. Lv X. Wang Y. Xun Z. Liu Z. (2014). The cysteine protease CEP1, a key executor involved in tapetal programmed cell death, regulates pollen development in Arabidopsis. *Plant Cell* 26 2939–2961. 10.1105/tpc.114.127282 25035401 PMC4145124

[B57] Zhang L. Wang M. Li N. Wang H. Qiu P. Pei L. (2018). Long noncoding RNAs involve in resistance to *Verticillium dahliae*, a fungal disease in cotton. *Plant Biotechnol. J.* 16 1172–1185. 10.1111/pbi.12861 29149461 PMC5978870

[B58] Zhang T. Hu Y. Jiang W. Fang L. Guan X. Chen J. (2015). Sequencing of allotetraploid cotton (*Gossypium hirsutum* L. acc. TM-1) provides a resource for fiber improvement. *Nat. Biotechnol.* 33 531–537. 10.1038/nbt.3207 25893781

[B59] Zhang X. M. Wang Y. Lv X. M. Li H. Sun P. Lu H. (2009). NtCP56, a new cysteine protease in *Nicotiana tabacum* L., involved in pollen grain development. *J. Exp. Bot.* 60 1569–1577. 10.1093/jxb/erp022 19246592 PMC2671612

[B60] Zou Z. Huang Q. Xie G. Yang L. (2018). Genome-wide comparative analysis of papain-like cysteine protease family genes in castor bean and physic nut. *Sci. Rep.* 8:331. 10.1038/s41598-017-18760-6 29321580 PMC5762910

[B61] Zou Z. Xie G. Yang L. (2017). Papain-like cysteine protease encoding genes in rubber (*Hevea brasiliensis*): comparative genomics, phylogenetic, and transcriptional profiling analysis. *Planta* 246 999–1018. 10.1007/s00425-017-2739-z 28752264

