# Supplementary material for: Genome-Wide Identification of Papain-Like Cysteine Proteases in Gossypium hirsutum and Functional Characterization in Response to Verticillium dahliae
Source: Front Plant Sci. 2019 Feb 20;10:134. doi: 10.3389/fpls.2019.00134 (PMC6391353; doi:10.3389/fpls.2019.00134)
Supplement: Supplementary file 1 [file Data_Sheet_1.docx]

Supplementary Material

**Genome-wide identification of papain-like cysteine protease family in *G. hirsutum* and functional characteration in response to *V.dahliae***

**Authors:** Shuqin Zhang, Zhongping Xu, Heng Sun, Longqing Sun, Muhammad Shaban, Xiyan Yang, Longfu Zhu*

* **Correspondence Author:** Longfu Zhu, National Key Laboratory of Crop Genetic Improvement, Huazhong Agricultural University, Wuhan 430070, Hubei, China. Tel: +86 027 87283955; E-mail: [lfzhu@mail.hzau.edu.cn](mailto:lfzhu@mail.hzau.edu.cn).


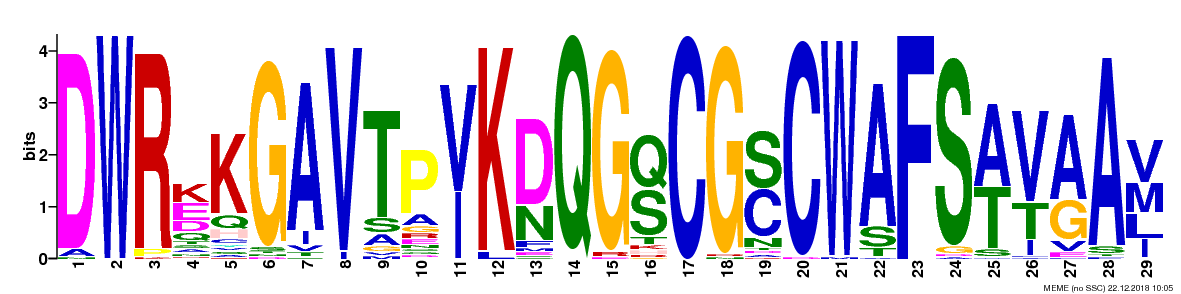


Motif1


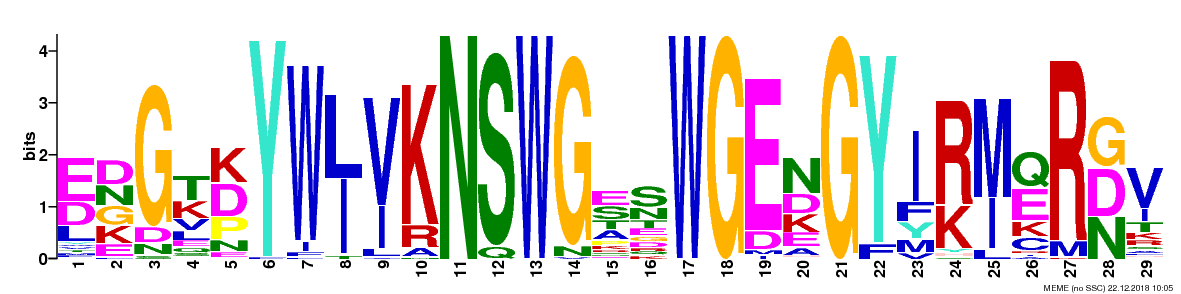


Motif2


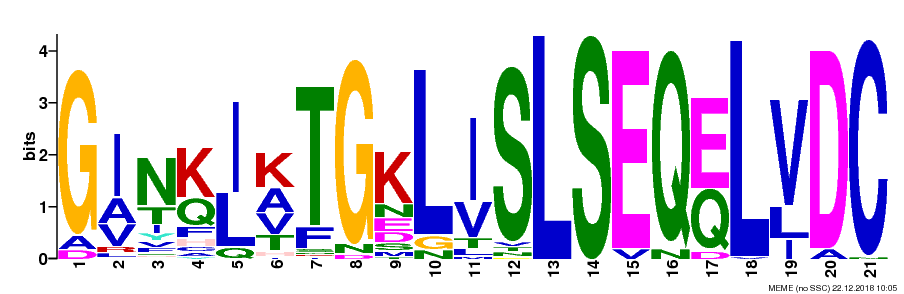


Motif3


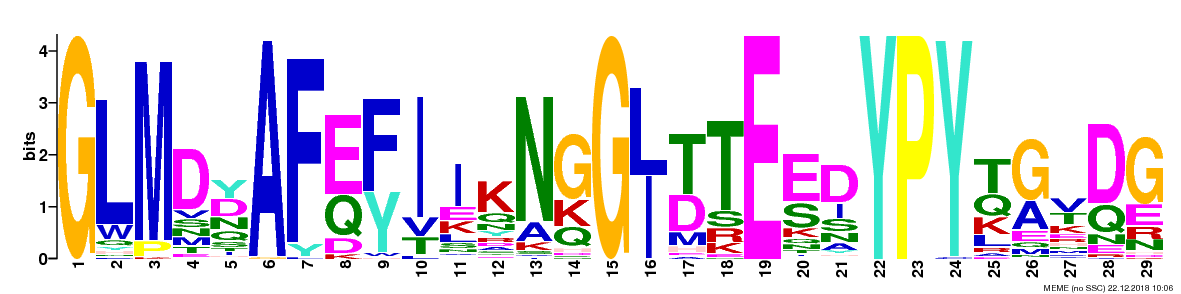


Motif4


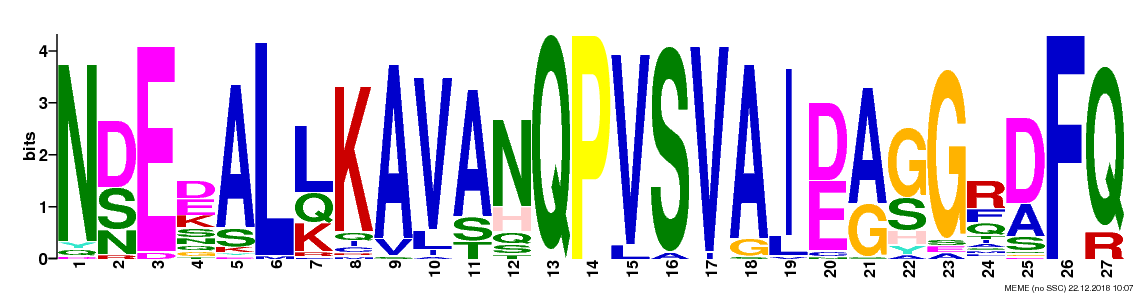


Motif5


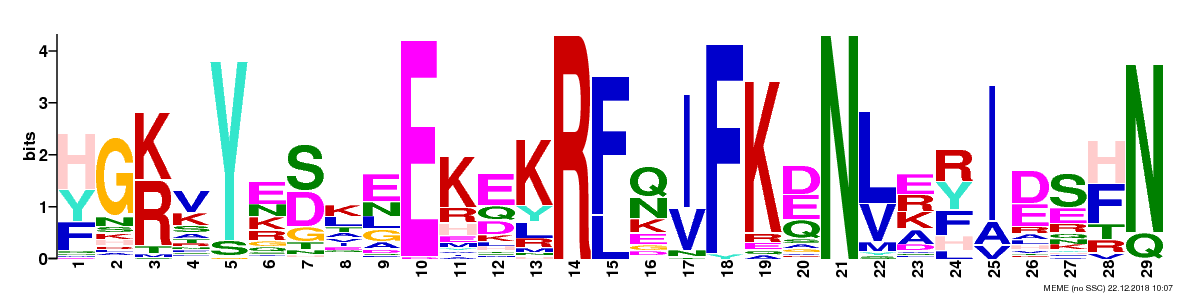


Motif6


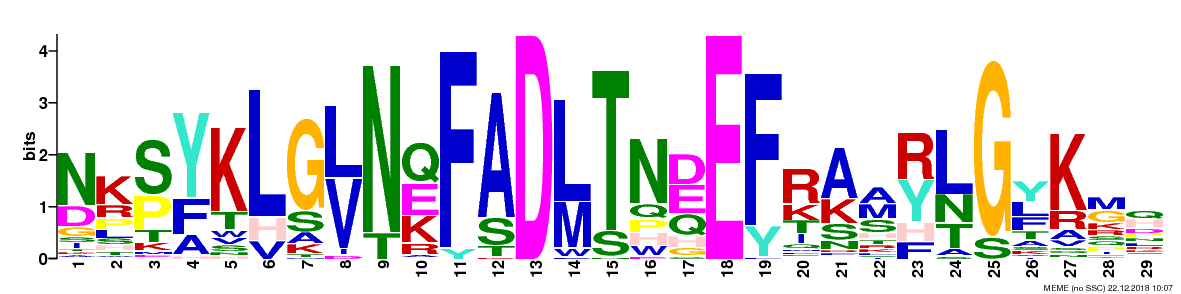


Motif7


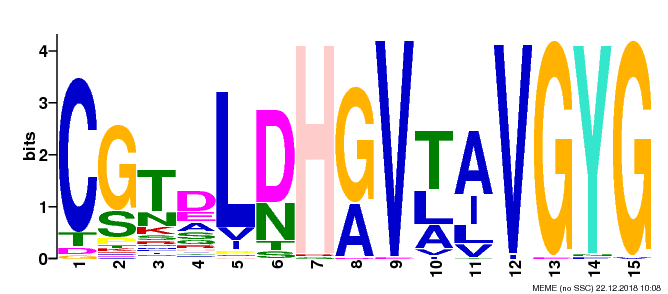


Motif8


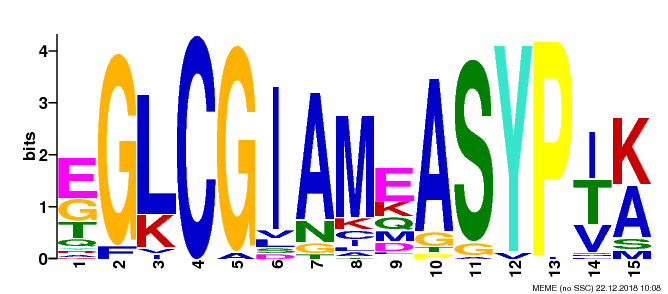


Motif9

Motif9


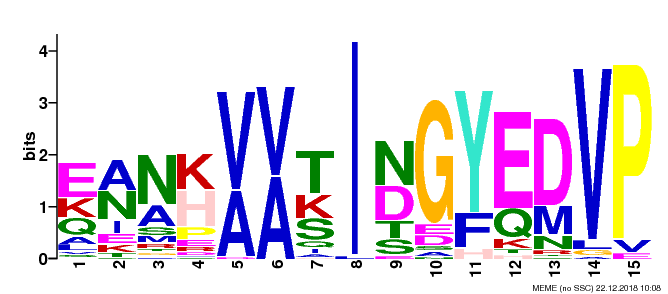


Motif10


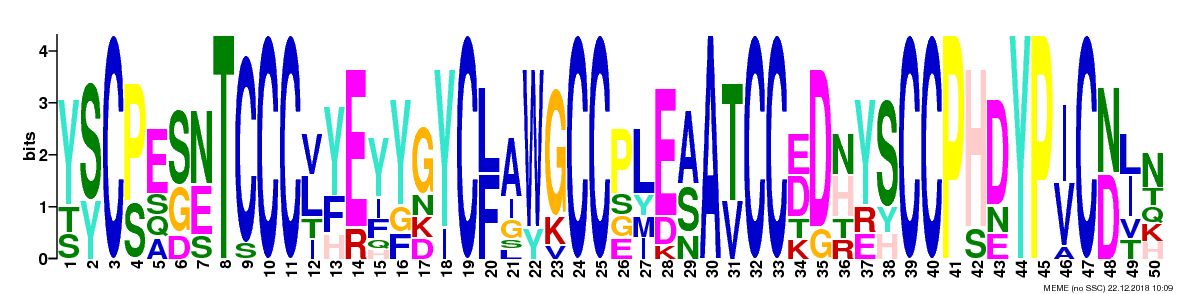


Motif11


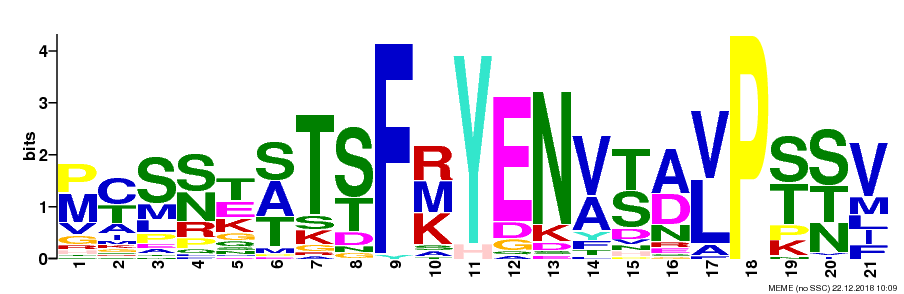


Motif12


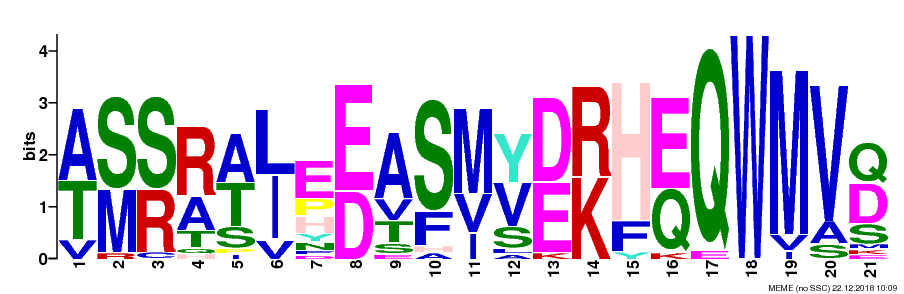


Motif13


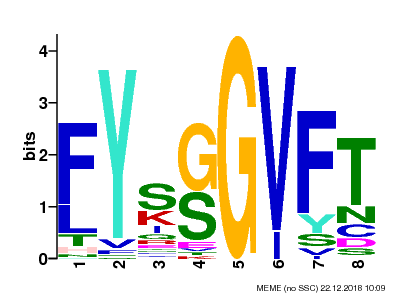


Motif14


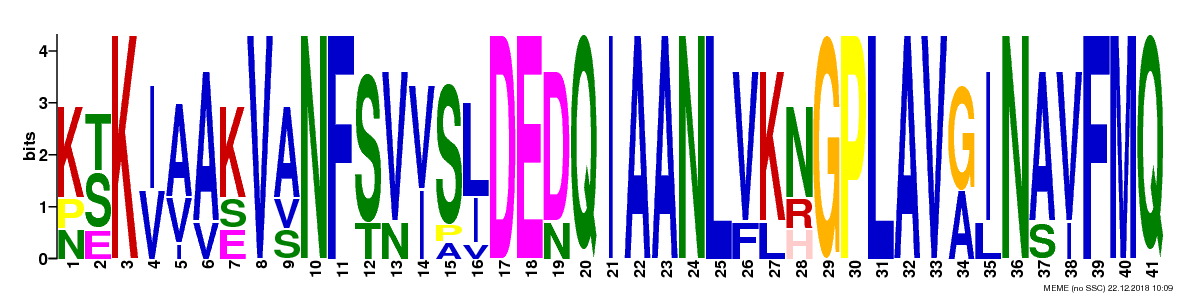


Motif15

**Supplementary Figure 1.** Sequence logo of different motifs identified in the PLCPs family.


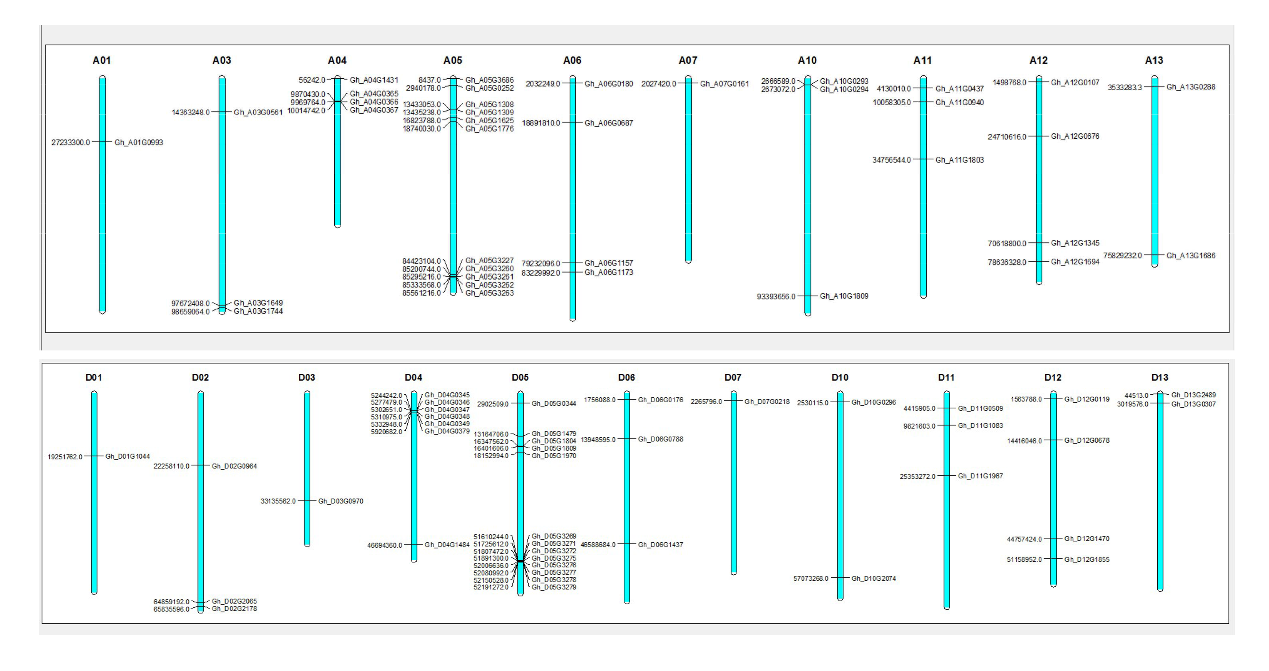


**Supplementary Figure 2.** Chromosomal distribution of *PLCP* genes on *G.hirsutum* chromosomes. The chromosome number (A1–D13) was shown on the top of each chromosome. The putative *PLCP* genes are shown on chromosomes 1–13 and from top to bottom. Green bars represent physical maps. Black lines on green bars indicate the locations of *PLCP* genes in each physical map. The scale is in megabases (Mb).


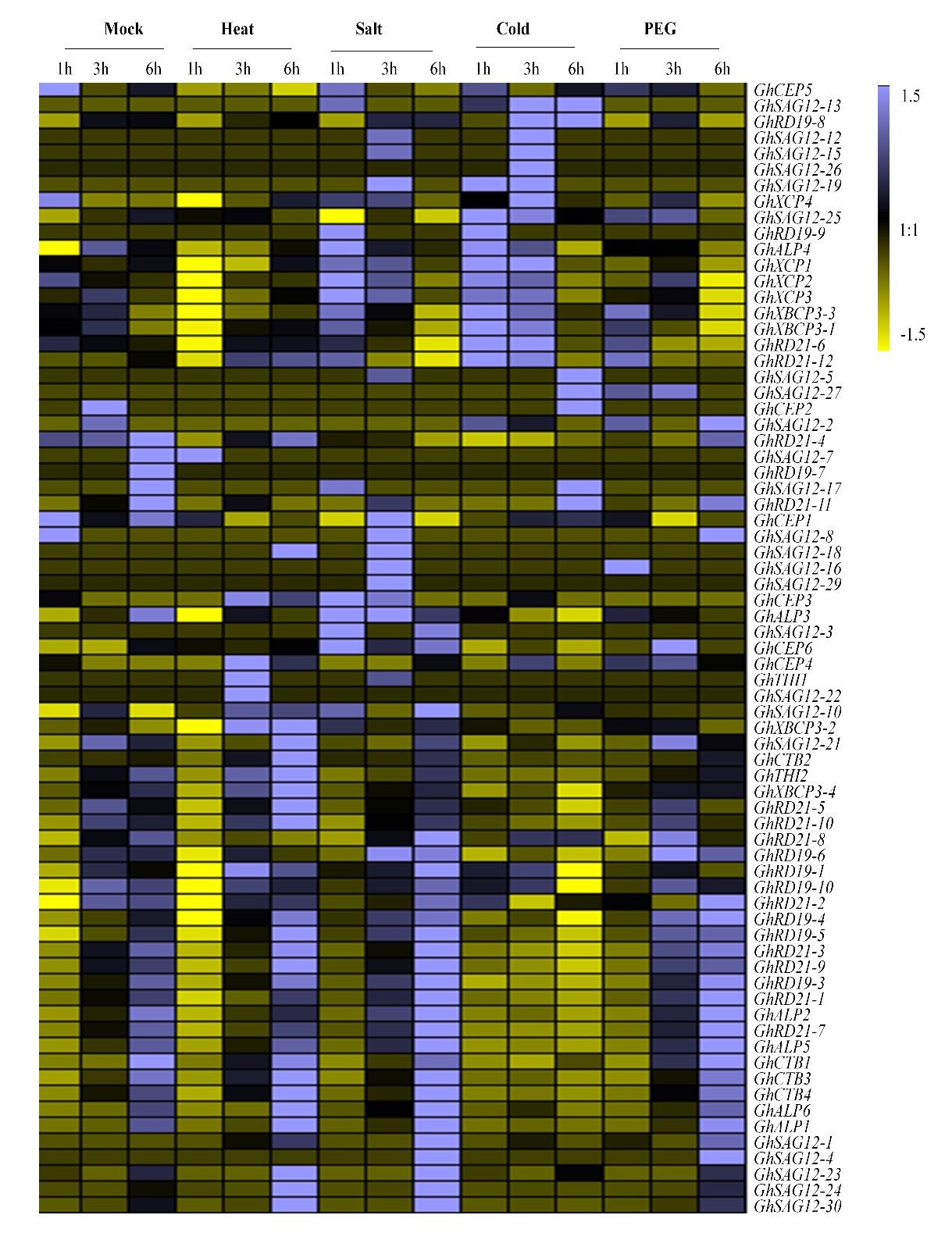


**Supplementary Figure 3.** Expression profile of *PLCP* genes in response to different abiotic stresses. Color scale denotes FPKM normalized by Genesis software.
